# Development of ABE and AKBE base editors in watermelon

**DOI:** 10.1093/hr/uhae123

**Published:** 2024-04-23

**Authors:** Dong Wang, Tao Zhu, Chunyu Liu, Yani Chen, Shujuan Tian, Chunhui Tian, Peng Gao, Shi Liu, Man Liu, Jiafa Wang, Xian Zhang, Feishi Luan, Li Yuan

**Affiliations:** State Key Laboratory of Crop Stress Resistance and High-Efficiency Production, College of Horticulture, Northwest A&F University, Yangling 712100, Shaanxi, China; State Key Laboratory of Crop Stress Resistance and High-Efficiency Production, College of Horticulture, Northwest A&F University, Yangling 712100, Shaanxi, China; State Key Laboratory of Crop Stress Resistance and High-Efficiency Production, College of Horticulture, Northwest A&F University, Yangling 712100, Shaanxi, China; State Key Laboratory of Crop Stress Resistance and High-Efficiency Production, College of Horticulture, Northwest A&F University, Yangling 712100, Shaanxi, China; State Key Laboratory of Crop Stress Resistance and High-Efficiency Production, College of Horticulture, Northwest A&F University, Yangling 712100, Shaanxi, China; State Key Laboratory of Crop Stress Resistance and High-Efficiency Production, College of Horticulture, Northwest A&F University, Yangling 712100, Shaanxi, China; Key Laboratory of Biology and Genetic Improvement of Horticulture Crops (Northeast Region), Ministry of Agriculture and Rural Affairs, College of Horticulture and Landscape Architecture, Northeast Agricultural University, 150030, Harbin, China; Key Laboratory of Biology and Genetic Improvement of Horticulture Crops (Northeast Region), Ministry of Agriculture and Rural Affairs, College of Horticulture and Landscape Architecture, Northeast Agricultural University, 150030, Harbin, China; State Key Laboratory of Crop Stress Resistance and High-Efficiency Production, College of Horticulture, Northwest A&F University, Yangling 712100, Shaanxi, China; State Key Laboratory of Crop Stress Resistance and High-Efficiency Production, College of Horticulture, Northwest A&F University, Yangling 712100, Shaanxi, China; State Key Laboratory of Crop Stress Resistance and High-Efficiency Production, College of Horticulture, Northwest A&F University, Yangling 712100, Shaanxi, China; Key Laboratory of Biology and Genetic Improvement of Horticulture Crops (Northeast Region), Ministry of Agriculture and Rural Affairs, College of Horticulture and Landscape Architecture, Northeast Agricultural University, 150030, Harbin, China; State Key Laboratory of Crop Stress Resistance and High-Efficiency Production, College of Horticulture, Northwest A&F University, Yangling 712100, Shaanxi, China

Dear Editor.

Watermelon (*Citrullus lanatus*), a popular and nutritionally valuable fruit, holds great appeal for consumers. Base editors represent cutting-edge genome-editing tools with tremendous potential not only for fundamental gene function characterization but also, more significantly, for advancing molecular breeding in crops. Although watermelon CBE (cytosine base editor) and CGBE (C-to-G base editor), capable of facilitating C-to-T/G/A, has been previously reported [1, 2], base editing specifically targeting adenine in watermelon has not been documented. In this study, we have developed watermelon BEs to implement different forms of adenine editing centered around TadA8e deaminase.

The primary role of ABE (adenine base editor) is to facilitate A:T-to-G:C base substitutions. In an effort to develop ABE in watermelon, we employed three distinct promoters, AtUbi, 2x35S, and AtRps5A, which have a relevant study in our watermelon cytosine editing [2], to orchestrate the expression of fusion proteins incorporating *Arabidopsis* codon-optimized TadA8e-nCas9 (D10A), denoted as U-ABE, S-ABE, and R-ABE, respectively (Fig. 1A). To assess the efficacy of these ABEs, we devised four sgRNAs targeting *ClALS1*, *ClACC*, and *ClFT* genes. Sanger sequencing of T_0_-edited plants revealed that S-ABE and R-ABE induced A:T-to-G:C editing to varying degrees across all four target sites, while U-ABE exhibited a lower extent of substitutions only in sgRNA1 (Fig. 1B and C). Exemplifying the results, the edited plants of R-ABE typically exhibit higher A:T-to-G:C base conversion efficiency and T0 generation editing efficiency; as a typical example, the edited plants of R-ABE typically exhibit higher A:T-to-G:C base conversion efficiency and deep sequencing data corresponding to sgRNA1 showcased that the Rps5A-promoted ABE (98.1%) outperformed S-ABE (52.1%) and U-ABE (23.0%) in terms of A:T-to-G:C base conversion efficiency at the A5 position (Fig. 1B and D). These findings underscore that the Rps5A promoter may be more favorable for A:T-to-G:C base substitutions in watermelon.

**Figure 1:**
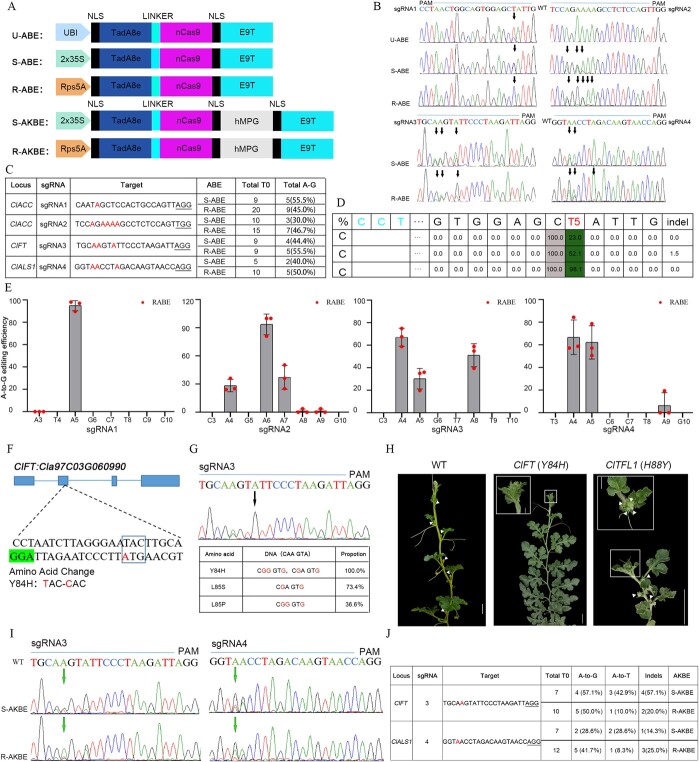
Development of adenine base editors in watermelon. **A** Schematic representation of A-to-G and A-to-K editors. **B** Representative sequence chromatograms demonstrating A:T-to-G:C base editing in sgRNA1–4 using U-ABE, S-ABE, and R-ABE. Arrows emphasize the efficiency of the A:T-to-G:C conversion. **C** Genotyping results of T_0_ edited plants using S-ABE and R-ABE. **D** Deep sequencing results revealing nucleotide changes caused by ABEs in watermelon T0 lines with sgRNA1, while the PAM sequence is highlighted. **E** Dot graph illustrating the A:T-to-G:C conversion frequencies of R-ABE for sgRNA1–4. Each data point represents one biological replicate at each target site. **F** Illustrations depicting the expected amino acid change caused by the A:T-to-G:C conversion in *ClFT*. **G** Genotype results of *ClFT* T_0_ plants using Sanger sequencing and Hi-TOM. **H** Comparison of the flowering time between a *ClFT* base-edited T_0_ plant generated by R-ABE and a *ClTFL1* base-edited T_0_ plant. Scale bar: 10 cm (the scale bars for enlarged images is 1 cm). **I** Examples of Sanger sequencing chromatograms for AKBEs in T_0_ plants with A:T-to-T:A base editing in sgRNA3 and sgRNA4. Arrows highlight the efficiency of the A:T-to-T:A conversion. **J** Genotyping results of T_0_ plants edited using S-AKBE and R-AKBE.

Additionally, the results revealed substantial variations in base conversion efficiencies at different A sites. This variability may be due to bases toward the target A site. To elucidate the editing preferences of R-ABE in watermelon more comprehensively, we conducted a statistical analysis of the results, as depicted in Fig. 1E. Remarkably, R-ABE demonstrated efficiently A:T-to-G:C substitutions at positions A4-A8 (base conversion efficiency exceeding 10%, indicating effective editing). Intriguingly, a notable decrease in efficiency was observed at the second A site when the edited sequence was AA, aligning with findings from a previous study [3]. Furthermore, among the 54 T_0_ plants generated by R-ABE, 26 plants demonstrated successful expected substitutions (Fig. 1C), resulting in a T0 editing efficiency of 48.1% (26/54, edited plants/total T_0_ transgenic plants). These findings further underscore the efficiency and feasibility of R-ABE in effecting A:T-to-G:C substitutions in watermelon.

The flowering process represents a pivotal developmental stage essential for ensuring reproductive success. Two homologous proteins, TFL1 and FT, play crucial roles in controlling flowering by influencing the timing and location of flower formation [4]. It has been proposed that the Y85H mutation in the AtFT (Arabidopsis FLOWERING LOCUS T) protein switches its function to that of the AtTFL1 (TERMINAL FLOWER 1) protein [5]. Sequence alignment of homologous proteins revealed that AtFT (Y85) and ClFT (Y84) have relatively conserved amino acids near the mutation site. To test whether the ClFT protein with the Y84H mutation might exhibit a function similar to ClTFL1, a crucial target was designed to achieve the Y84H mutation in ClFT (Fig. 1F). The mutation analysis indicated that five out of the T_0_ plants exhibited A-to-G editing, resulting in a T0 editing efficiency of 55.6% (5/9), as shown in Fig. 1C. Additionally, the *ClFT (Y84H)* transgenic plants led to three distinct types of amino acid substitutions, as depicted in Fig. 1G. The edited plants carrying the Y84H mutations exhibited a flowerless trait (Fig. 1H), this outcome may be attributed to the mutation, affecting ClFT ability to induce downstream genes essential for flower bud differentiation. Given that the pure segregation of progeny plants was hindered by the suppression of reproductive growth, it became essential to acquire a broader array of mutant plants for analysis. This step was crucial to identify which specific mutation was responsible for the development of the flowerless trait in watermelon. Conversely, *ClTFL1* (*H88Y*) edited plants obtained by CBE displayed the trait of early flowering (Fig. 1H). In summary, the edited *ClFT* (*Y84H*) and *ClTFL1* (*H88Y*) exhibited opposite phenotypes, suggesting an internal switch in the roles of vegetative and reproductive genes. This demonstrates the significant potential of R-ABE in watermelon breeding.

However, the implementation of existing base editor context did not fully meet the requirements for watermelon breeding. We aimed to explore alternative adenine mutation forms, such as A:T-to-T:A and A:T-to-C:G. MPG (the N-methylpurine DNA glycosylase protein), acting as hypoxanthine excision protein, was considered for its potential role in facilitating other types of adenine mutations. Initial experiments utilizing *Arabidopsis* MPG indicated an absence of additional adenine edits, aside from A:T-to-G:C. Conversely, it has been documented that human MPG (hMPG), featuring seven amino-acid mutations, can effectively achieve A-to-Y (Y = T/C) base substitutions [6]. We opted to replace our initial AtMPG with *Arabidopsis* codon-optimized hMPG, incorporating the necessary mutations. The resulting editors were termed as S-AKBE (2x35S) and R-AKBE (Rps5A), respectively (Fig. 1A). In the evaluation of four sgRNAs, sgRNA1 and 2 exclusively induced A:T-to-G:C base mutations; however, in sgRNA3 (position A5) and sgRNA4 (position A4), S-AKBE and R-AKBE produced A-to-K (T/G) editing with varying efficiencies (Fig. 1I). In contrast to the A-to-Y preferences observed in human cells, our AKBE base editors demonstrated greater preference with A-to-K mutations, aligning with recent findings in rice and tomato [7–9]. The results revealed that S-AKBE exhibited significantly higher A:T-to-C:G editing efficiency than R-AKBE in T_0_ plants (Fig. 1J), which is in contrast to the preference for the Rps5A promoter in ABE. Consequently, S-AKBE emerged as a valuable adenine editing tool in watermelon, achieving a gene editing efficiency in the T_0_ generation of 35.7% (5/14) (Fig. 1J).

However, the T_0_ plants edited through the S-AKBE are likely to result in A-to-G/T chimeras. Notably, all plants exhibiting A:T-to-T:A modifications harbored A:T-to-G:C base substitutions, and the editing efficiency for the T_0_ generation plants with the A:T-to-G:C mutation reached 42.9% (6/14), surpassing that of the A:T-to-T:A edited plants (Fig. 1J). When addressing chimeric mutations introduced by AKBE, it is imperative to pinpoint the precise target mutations and assess the isolated homozygous individuals through both phenotypic observations and molecular analyses. Additionally, we noted that the T_0_ generation plants derived from S-AKBE exhibited a range of indels (5/14, 35.7%), including those that did not achieve A:T-to-T:A editing (Fig. 1J). This phenomenon could stem from the action of adenine deaminase, which converts adenine into inosine (I) upon targeting a site for deamination, while the mutated hMPG enzyme removes hypoxanthine, leading to the formation of an apurinic-apyrimidinic (AP) site. This, in turn, can increase the likelihood of indel formation during the DNA repair process. Consequently, devising strategies to enhance the editing efficiency for A:T-to-T:A substitutions and to minimize the occurrence of indels represents a key focus for future research. Despite the need for further refinement, the unique capability of S-AKBE to facilitate A:T-to-T:A substitutions underscores its significant potential for advancing watermelon base editing technologies.

So far, we have successfully established efficient ABE and AKBE editors in watermelon. We employed R-ABE to generate *ClFT (Y84H)* mutant plants exhibiting a flowerless phenotype, and the accomplishment of A:T-to-T:A mutation with a remarkable T0 generation efficiency of 35.7% using S-AKBE. This breakthrough enables us to achieve diverse forms of base conversion in watermelon. Meanwhile, research involving base editing techniques extends beyond precise base editing, encompassing the exploration of unknown SNPs that may impact traits. This is achieved by inducing mutations throughout the entire coding region of the target gene using base editors, underscoring its significance [10]. The initial implementation of adenine base editors in watermelon has facilitated the creation of dual-base editors and established a solid groundwork for our future studies on saturation mutations. Looking ahead, further optimization of the existing base editing system and the exploration of additional target genes hold the potential to significantly enhance watermelon molecular breeding processes.

## Acknowledgements

We thank Dr Huanbin Zhou for his technical advisement and support. This work was supported by the Key Program of the National Natural Science Foundation of China (U23A20208), the National Youth Talent Program (A279021801), the National Key Research and Development Program of China (2023YFE0206900), the National Natural Science Foundation of China (32372734), Luoyang Major Science and Technology Tackling Key Issues Project (Public Announcement and Leadership, 2301024A), Earmarked Fund for China Agriculture Research System (CARS-25), Key-Area R&D Program of Guangdong Province (2022B0202060001), Key R&D Program of Shaanxi Province (2023-YBNY-008, 2024NC-YBXM-032), and the Natural Science Foundation of Shaanxi Province (2022JM-112).

## Author contributions

L.Y., F.L., and X.Z. conceived the study. D.W., T.Z., C.L., Y.C., S.T. C.T., P.G., S.L., M.L., and J.W. executed the experiments. L.Y. and D.W. wrote the manuscript.

## Data availability

The plasmid used in this study will be available at Addgene, and the supplementary vector sequences, primers, and methods generated during this study are available in the Figshare database under the accession https://figshare.com/s/c314f1eb66982d91bbea. The additional data related to this paper may be requested from the authors.

## Conflict of interest statement

The authors declare no competing financial interests.
